# Effective gene silencing using type I–E CRISPR system in the multiploid, radiation-resistant bacterium *Deinococcus radiodurans*

**DOI:** 10.1128/spectrum.05204-22

**Published:** 2023-09-06

**Authors:** Chitra S. Misra, Neha Pandey, Deepti Appukuttan, Devashish Rath

**Affiliations:** 1 Applied Genomics Section, Bio-Science Group, Bhabha Atomic Research Centre, Mumbai, Maharashtra, India; 2 Life Sciences, Mumbai University, Mumbai, Maharashtra, India; 3 Chemical Engineering Department, IIT Bombay, Mumbai, Maharashtra, India; 4 Homi Bhabha National Institute, Mumbai, Maharashtra, India; University of Manitoba, Winnipeg, Manitoba, Canada

**Keywords:** CRISPRi, *Deinococcus radiodurans*, Cascade, gene silencing

## Abstract

The extremely radiation-resistant bacterium, *Deinococcus radiodurans*, is a microbe of importance, both, for studying stress tolerance mechanisms and as a chassis for industrial biotechnology. However, the molecular tools available for use in this organism continue to be limiting, with its multiploid genome presenting an additional challenge. In view of this, the clustered regularly interspaced short palindromic repeat (CRISPR)-Cas tools provide a large repertoire of applications for gene manipulation. We show the utility of the type I-E Cascade system for knocking down gene expression in this organism. A single-vector system was designed for the expression of the Cascade components as well as the crRNA. The type I-E Cascade system was better tolerated than the type II-A dCas9 system in *D. radiodurans*. An assayable acid phosphatase gene, *phoN* integrated into the genome of this organism could be knocked down to 10% of its activity using the Cascade system. Cascade-based knockdown of *ssb*, a gene important for radiation resistance resulted in poor recovery post-irradiation. Targeting the Radiation and Desiccation Response Motif (RDRM), upstream of the *ssb*, prevented de-repression of its expression upon radiation exposure. In addition to this, multi-locus targeting was demonstrated on the deinococcal genome, by knocking down both *phoN* and *ssb* expression simultaneously. The programmable CRISPR interference tool developed in this study will facilitate the study of essential genes, hypothetical genes, and *cis*-elements involved in radiation response as well as enable metabolic engineering in this organism. Further, the tool can be extended for implementing high-throughput approaches in such studies.

**IMPORTANCE:**

*Deinococcus radiodurans* is a microbe that exhibits a very high degree of radiation resistance. In addition, it is also identified as an organism of industrial importance. We report the development of a gene-knockdown system in this organism by engineering a type I-E clustered regularly interspaced short palindromic repeat (CRISPR)-Cascade system. We used this system to silence an assayable acid phosphatase gene, *phoN* to 10% of its activity. The study further shows the application of the Cascade system to target an essential gene *ssb*, that caused poor recovery from radiation. We demonstrate the utility of CRISPR-Cascade to study the role of a regulatory *cis*-element in radiation response as well as for multi-gene silencing. This easy-to-implement CRISPR interference system would provide an effective tool for better understanding of complex phenomena such as radiation response in *D. radiodurans* and may also enhance the potential of this microbe for industrial application.

## INTRODUCTION

As an extremophile, *Deinococcus radiodurans* (*D. radiodurans*) is well-known for its high tolerance to damage caused by ionizing and UV radiation (1). The bacterium is also extremely resistant to desiccation (2) and the vacuum pressure of space (3). Studies over the years have attributed various mechanisms including efficient protection of its proteome and efficient repair of damaged DNA to contribute to its survival of extreme conditions (1, 4). Apart from serving as a model system to understand extreme stress tolerance, these characteristics have made *D. radiodurans* an attractive organism for biotechnological applications such as bioremediation (5), production of unique pigments (6), and production of small molecules and metabolites (7, 8).

With the availability of defined promoters for inducible expression of genes (9, 10), selectable markers (11 – 13), the development of shuttle plasmids (14, 15), recombineering (16), and conjugation systems (17), the toolkit for the genetic manipulation of this important organism has been expanding. Importantly, none of these methods are suitable for targeting essential genes. It is conceivable to use an inducible promoter to conditionally complement a chromosomal gene disruption, as has been reported for *ssb* (18). However, such a strategy would not only remove the gene from its chromosomal context but maintaining the native expression level of the target gene would also be a major challenge in the conditional mutant. In most cases, these methods are not easy to use, likely due to the presence of active restriction-modification systems known to digest exogenous DNA. Genetic modifications are also difficult to achieve owing to the polyploid nature of *D. radiodurans*, which can have up to 10 copies of its genome (19).

Clustered regularly interspaced short palindromic repeat (CRISPR)-based technology has revolutionized targeted gene manipulation in a multitude of organisms (20). CRISPR editing systems have typically used Cas endonuclease and a paired guide RNA (gRNA). Most gRNAs consist of a fixed sequence derived from the CRISPR repeat and a variable “spacer” region complementary to the targeted locus (21). The gRNA-Cas endonuclease complex interacts with the target locus based on the complementary binding of the spacer region of gRNA to the target. Additionally, this interaction requires the presence of a small three to five nucleotide long protospacer adjacent motif (PAM) in the target locus. After the recognition and binding of the gRNA-Cas endonuclease complex, a double-stranded break (DSB) is generated within the target DNA (21). The repair of DSB can proceed via nonhomologous end joining or via homology-directed repair. For homology-directed repair, a donor DNA, with homology to the region surrounding the DSB needs to be provided. Both these mechanisms have been exploited for gene editing in many organisms (22).

CRISPR systems have also been modified to achieve transcriptional repression, a technology referred to as “CRISPRi.” CRISPR interference (CRISPRi) was first demonstrated in *Escherichia coli* (*E. coli*) and mammalian cells, where a type II CRISPR system was used (23). For transcriptional repression, a mutated version of Cas endonuclease (dead Cas9 or dCas9) that had lost the nuclease function but retained the ability to bind to the target was used to target the promoter or the ORF region of a gene by use of gRNAs designed for the purpose. Binding of the dCas9-gRNA complex to the promoter or the ORF region caused a steric hindrance for the RNA polymerase, thus blocking transcription initiation or elongation (23). Subsequently, Rath et al. (24) repurposed a type I–E system for CRISPRi in *E. coli*. Type I-E CRISPR system forms a ribonucleoprotein complex (Cascade) consisting of crRNA and five Cas proteins (Cse1, Cse2, Cas7, Cas5, and Cas6e). Upon target binding, Cascade recruits a nuclease, Cas3, which degrades the target DNA. For achieving transcriptional repression, Cascade was guided with engineered crRNAs to bind to the promoter or the ORF of the target gene, while the gene for Cas3 was deleted to prevent cleavage of the target DNA (24). Subsequent to these initial reports CRISPRi has been implemented in a variety of other microbial species (25) (and references therein).

As compared to gene editing, CRISPRi has its own advantages. It is relatively easy to engineer for it does not require the presence of a donor template as in the case of editing by homology-directed repair. It allows for regulation of the level of target gene expression with the added advantage that the regulation can be reversed. The CRISPRi can be easily converted to a CRISPR activation (CRISPRa) framework by fusing activator domains to Cas proteins to drive transcriptional activation from a desired locus (26, 27). Most importantly, it facilitates the study of essential genes which cannot be deleted/mutated or for which it is difficult to obtain conditional mutants. So far, CRISPR-based genetic manipulation systems have not been reported in *D. radiodurans*. In this study, we report the design and development of a type I-E Cascade-based CRISPRi platform for transcriptional repression of gene expression in *D. radiodurans*.

## MATERIALS AND METHODS

### Strains and growth conditions

*D. radiodurans R1* and *E. coli* JM109 and DH5α strains were used in the study. *E. coli* strains were grown in Luria Bertani (LB) broth. The LB medium was supplemented with appropriate antibiotics (carbenicillin 100 µg mL^−1^ and spectinomycin 40 µg mL^−1^), wherever required. To prepare solid medium, 1.5% agar was included in the medium. *E. coli* cultures were grown at 37°C with shaking at 120 rpm. *D. radiodurans* was grown at 32°C in tryptone, glucose, yeast extract (TGY) with appropriate antibiotics (chloramphenicol 3 µg mL^−1^, spectinomycin 75 µg mL^−1^, and kanamycin 8 µg mL^−1^), wherever needed. The amylase activity of the integrated *Deinococcus phoN* + clones was tested on TY starch agar plates as described earlier (28). List of strains used in this study is given in Table S1.

### Recombinant DNA methods

For all recombinant DNA procedures, standard methods as described (29) were followed. *E. coli* strains, JM109 and DH5α, were used for vector constructions. For deinococcal work, the procedures are described in the appropriate sections below.

### Development of CRISPRi platform

The shuttle vectors pRAD1 or pVHS559 (30) were used for expression of the CRISPR systems in *D. radiodurans* (Table S1). The *dcas9* ORF, codon-optimized for *Mycobacterium smegmatis*, was analyzed for codon usage in *D. radiodurans* using Graphical Codon Analyzer (https://bio.tools/gcua) (31). The *dcas9* originally cloned in the pSTKT vector was released by digestion with NdeI-BamHI and cloned into identical sites of the pRAD1 vector to generate pCRD1 (32).

**Fig 1:**
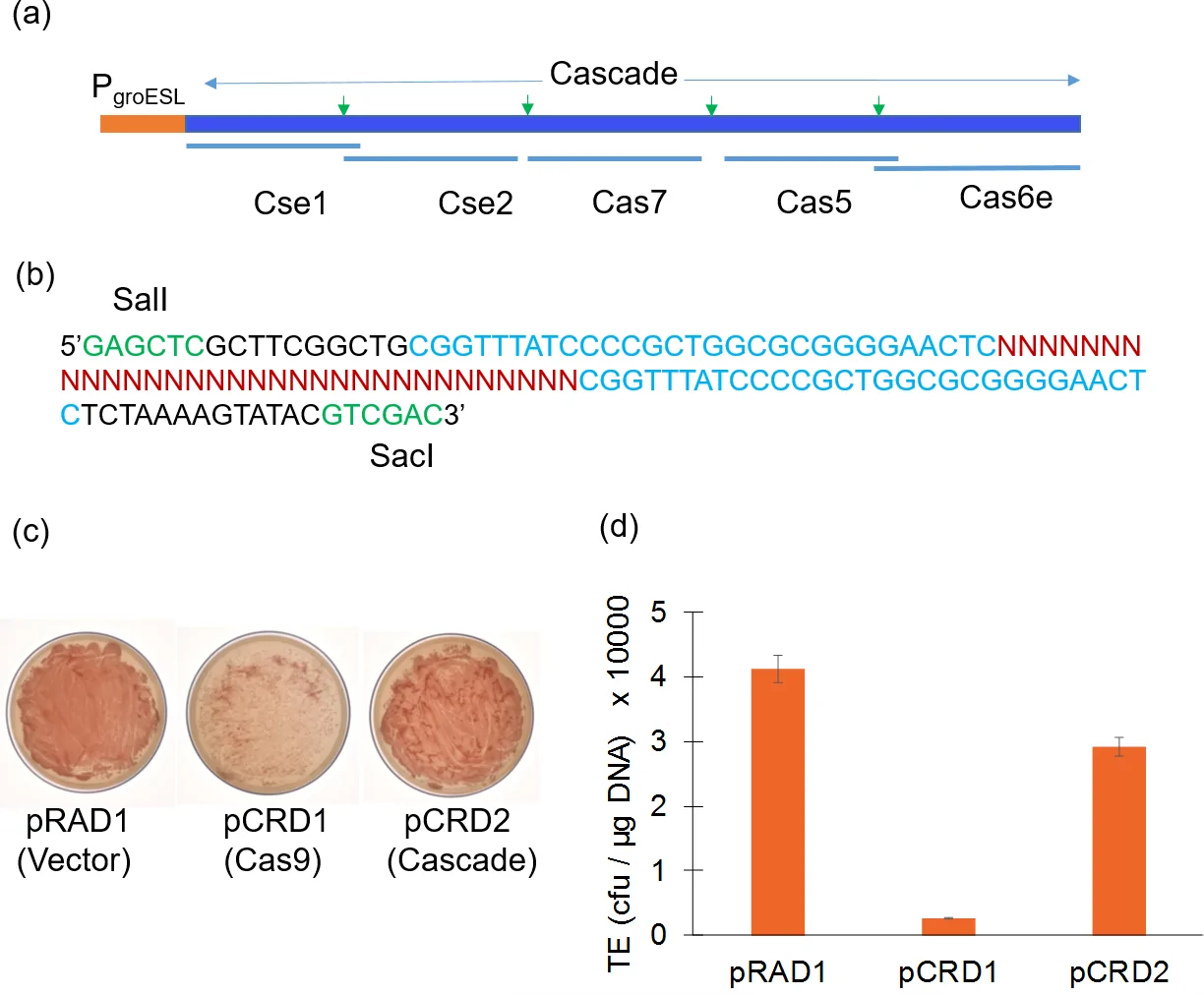
CRISPR-Cascade for gene silencing in *Deinococcus radiodurans*. Codon-optimized Cascade operon, with the overlapping nature of ORFs kept intact and Shine Dalgarno (green arrows) sequences retained (a). crRNA sequence showing repeat sequences (in blue) on either side of the targeting spacer (in maroon), with flanking restriction sites for cloning marked in green (b). Plasmids, pRAD1 or pCRD1 (pRAD1 expressing *dcas9*) or pCRD2 (pRAD1 expressing Cascade genes) were transformed into *D. radiodurans* and plated on agar plate containing chloramphenicol for selection (c). Transformation efficiency (TE) obtained with 1 µg of each plasmid (d).

The CRISPR type I-E Cascade operon from *E. coli* K-12 MG1655 was codon optimized for expression in *D. radiodurans* and synthesized along with the sequence coding for the crRNA. The synthesized sequence contained the crRNA sequence under the control of the constitutive promoter, P_groESL_, and the ORFs of the codon-optimized Cascade subunits. The Shine Dalgarno sequences for each of the Cascade ORFs remained unchanged while optimizing codon usage (Fig. 1a; Table S2). The crRNA was designed such that the repeat-spacer-repeat sequence was flanked by SacI-SalI sites for cloning spacers for defining new targets (Fig. 1b). P_groESL_ was PCR amplified from *D. radiodurans* and cloned in the BamHI-NdeI sites of pRAD1 (15) to generate pRA-gro. The synthesized Cascade-crRNA fragment was subsequently cloned between the NdeI-XhoI sites of this vector to generate pCRD2. Cascade-crRNA fragment was also cloned between the NdeI-XhoI sites of the pVHS559 (30) to generate pCRD3. This placed the Cascade operon expression under the inducible P_spac_ promoter. List of plasmids used in the study is given in Table S1.

### Phosphatase assays

Phosphatase assays were performed as described before (33). For whole-cell assays, cell density was equalized and the cells were incubated with the substrate, *p*-nitrophenol phosphate (*p*NPP), in acetate buffer (100 mM, pH 5.0) at 37°C for 1 h. The reaction was stopped by adding 0.2 N NaOH. The product, *p*-nitrophenol (*p*NP), formed due to phosphatase action was estimated by recording absorbance at 405 nm. Activity obtained in *D. radiodurans phoN+* strain was normalized against phosphatase activity obtained in wild-type strain to quantify the contribution of *phoN* alone. In addition to this, phosphatase activity was also determined in the gel by zymogram as described earlier (33). Briefly, the cells were lysed in non-reducing Laemlli’s buffer, and equal amount of protein was loaded onto gels. Protein estimation was done by modified Lowry’s method as described earlier (34). Post-electrophoretic separation, the gel was serially washed once with water and then twice with Tris buffer (100 mM, pH 8). The gel was subsequently developed using nitroblue tetrazolium-5-bromo-4-chloro-3-indolylphosphate mix in 100 mM Tris buffer pH 8.0. For assaying phosphatase activity on histochemical plates, phenolphthalein diphosphate (PDP) (1 mg mL^−1^) and methyl green (MG) (5 µg mL^−1^) were added to TGY agar.

### Integration of *phoN* into the deinococcal chromosome

To generate a stable easy-to-assay system for CRISPRi evaluation in *D. radiodurans*, *phoN*, coding for acid phosphatase from *Salmonella typhi*, was integrated into the chromosome. The recombinant plasmid pPN1 (33) was used as template for amplifying the deinococcal *groESL* promoter along with the *phoN* ORF (P*_groESL_ + phoN*). The integration plasmid, pGroES4Z, was used as the vector for the transport of the desired insert into the *D. radiodurans* genome (28). The plasmid pGroES4Z is an integration vector with a kanamycin cassette flanked by 5′ and 3′ segments of *amyE* gene, which, upon integration, replaces the wild-type *amyE* gene of *D. radiodurans* leading to loss of starch degrading ability. A 1.2 kb DNA fragment containing *phoN* downstream of deinococcal *groESL* promoter was PCR amplified from plasmid pPN1 using primer pairs P5/P6 and introduced into the XcmI restricted site of pGroES4Z, yielding a 9.4 kb recombinant plasmid, pGroES4ZN. A schematic representation of the cloning strategy is shown in Fig. S1. pGroES4ZN was used to transform *E. coli* DH5α and recombinants were selected on ampicillin plates to obtain *E. coli*-pGroES4ZN clones. The plasmids isolated from these cells were sequenced to rule out mutations and then transformed into *D. radiodurans* as described (33). The deinococcal recombinants selected on kanamycin were analyzed for integration/recombination event by diagnostic PCR with primers, Amy-1, Amy-2, Amy-3, and Amy-4. The integration was confirmed by DNA sequencing. The details of the primers used are given in Table S3.

Kanamycin-resistant clones obtained were screened for phosphatase activity by plating on media containing PDP-MG. Green-colored colonies obtained with the recombinant strain indicated phosphatase activity, while wild-type cells showed typical orange-colored colonies (Fig. S1b). Whole-cell *p*NPP phosphatase assay also confirmed the presence of the *phoN* activity (Table S4). The vector pGroES4Z integrates into the *Deinococcus* genome at the *amyE* locus and hence during a double recombination event, there is disruption of this gene, resulting in inability to degrade starch. The wild-type *D. radiodurans* and the integrated *Deinococcus phoN* + clones were analyzed for starch degradation on a starch agar plate. A typical result is shown in Fig. S1c. Wild-type *D. radiodurans* showed a halo zone around its growth on the starch agar plate when flooded with iodine solution. Under similar conditions, no halo zone was observed with the *Deinococcus phoN* + clone, indicating a disruption in the *amyE* gene (Fig. S1c). This analysis confirmed the integration of the *phoN* in the *amyE* locus of the *D. radiodurans* genome.

### Cloning and expression of crRNA targeting different loci on the deinococcal genome

To clone and express sequences coding for the crRNAs for different targets, 90–98 bases long oligonucleotides were synthesized with compatible overhangs for the SacI and SalI sites. The single-stranded oligos (100 µM) were annealed in annealing buffer (10 mM Tris HCL, 50 mM NaCl, and 1 mM EDTA) with gradual cooling from 94°C for 4 min, 75°C for 5 min, 65°C for 15 min to 25°C for 20 min. The annealed oligos were diluted 10 times and ligated into pCRD2 digested with SacI and SalI, and transformed into *E. coli* JM109. The transformants were selected on carbenicillin and screened by sequencing of the plasmids to confirm the presence of desired target sequence in the crRNA. Oligonucleotides used for cloning of crRNAs for different targets are listed in Table S3.

### Irradiation and growth curve

Cultures of *D. radiodurans* recombinants were grown overnight, re-suspended at final OD_600nm_ of 3.0 and exposed to Co-60 gamma source to a cumulative dose of 7 kGy (4.5 kGy/h). Cells suspended at similar optical density which were kept outside the irradiator served as control. The irradiated cells and control cells were subsequently washed in TGY and re-suspended in fresh media to achieve a final OD_600nm_ of 0.5. Cultures were grown for 3 h for post-irradiation recovery (PIR) before harvesting them to extract protein. The Ssb levels in the cells were determined by Western blot using anti-Ssb antibody (35). For determining growth kinetics, irradiated and control cells were inoculated into fresh media at a starting OD_600nm_ of 0.1 in microtitre plates. The plate was kept at 32°C, with shaking (200 rpm) in the plate reader (Infinite M Plex, Tecan Instruments) for acquiring absorbance reading at 600 nm at 30 min intervals for 36 h.

## RESULTS

### Development of CRISPRi system

A type II-A system from *Streptococcus pyogenes* has been widely adapted for CRISPRi as it involves a single protein, dCas9, and a cognate sgRNA. The codon frequency of *M. smegmatis* and *D. radiodurans* is similar owing to similarly rich GC content of their genome. The *dcas9* sequence codon-optimized for *M. smegmatis* (32) was tested *in silico* for codon usage in *D. radiodurans* and it was found suitable (Fig. S2). This sequence was cloned into pRAD1 under the constitutive promoter P_groESL_ to construct pCRD1. The transformation efficiency (TE) in *D. radiodurans* was about 16 times lower with pCRD1 than pRAD1 indicating a high level of toxicity for dCas9 (Fig. 1c and d). Cas9 is known to cause toxicity in several microbes where it has been applied (36 – 39). In view of the poor transformation efficiency obtained with the pCRD1 plasmid, we chose to implement the type I-E Cascade-based system from *E. coli* in *D. radiodurans*. The type I-E system consists of Cas1 and Cas2 (involved in adaptation), a complex of five Cas proteins (Cse1, Cse2, Cas7, Cas5, and Cas6e) named “Cascade,” which along with crRNA forms the surveillance complex, and Cas3 nuclease, which is recruited by Cascade after target recognition for cleavage of the target DNA (40). For implementing CRISPRi in *D. radiodurans*, we designed pCRD2, a single vector system that contained both a crRNA expression cassette and an operon coding for Cascade proteins (Fig. 1a and b) under control of the strong deinococcal constitutive promoter, P_groESL_. In parallel, an IPTG inducible system for Cascade expression was also designed by cloning the Cascade genes under the P_spac_ promoter in pCRD3. Cas1, Cas2, and the Cas3 nuclease were intentionally left out to ensure that the target DNA was not cleaved. In *D. radiodurans*, the transformation efficiency with pCRD2 was only 1.4-fold less than that of the parental vector pRAD1 (Fig. 1c and d) indicating better tolerance of the Cascade system than the dCas9 system in *D. radiodurans*. Similar results were obtained with pCRD3 (data not shown).

### CRISPRi for downregulation of *phoN* gene

**Fig 2:**
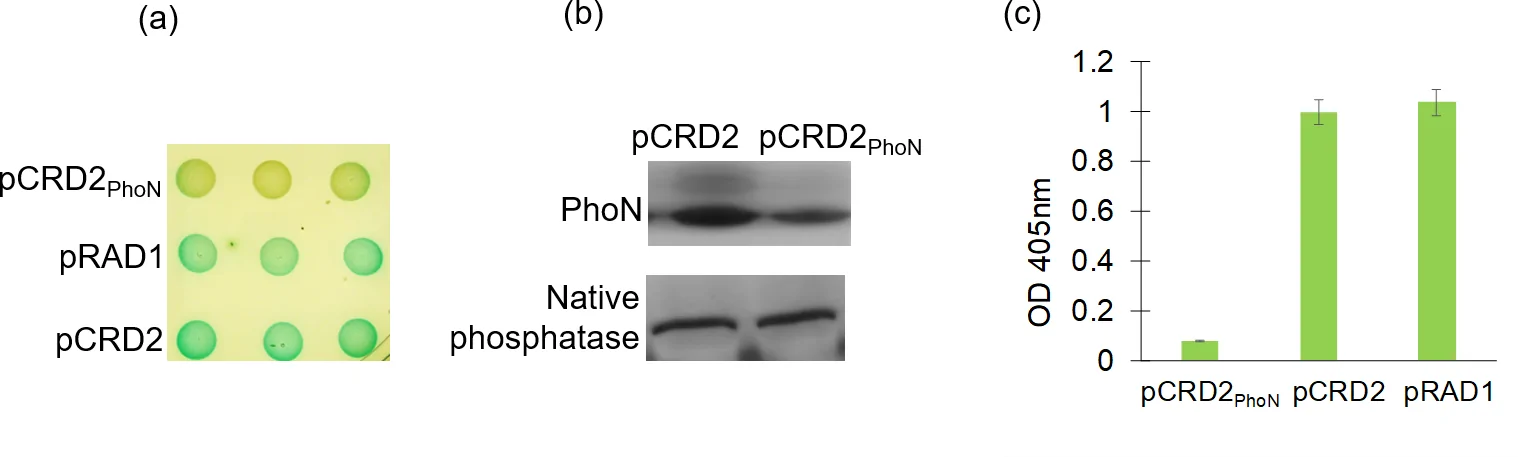
Phosphatase silencing using CRISPR-Cascade system in *D. radiodurans phoN+*. Phosphatase silencing in recombinants carrying pRAD1, or pCRD2 (pRAD1 expressing Cascade), or pCRD2_PhoN_ (pRAD1 expressing Cascade and spacer targeting *phoN*) was assayed by PDP-MG histochemical assays (a), zymogram (b), and biochemical assay (c). Phosphatase activity in *phoN+* cells was normalized against activity in wild-type cells.

The crRNA was designed to target the sequence immediately downstream of the *phoN* start codon on the chromosome. The sequence coding for the *phoN-*targeting crRNA was cloned in pCRD2 to generate, pCRD2_PhoN_ as well as in pCRD3 to generate pCRD3_PhoN_. The plasmids were individually transformed into *D. radiodurans phoN+* strain and phosphatase activity was screened in the transformants. Initial screening by patching of cells on histochemical plates showed reduced green coloration for recombinants carrying pCRD2_PhoN_, where Cascade expression was constitutive suggesting silencing of *phoN* while cells with pCRD2 showed intense green coloration. However, in recombinants carrying pCRD3_PhoN_, inducing Cascade expression by IPTG did not result in reduced PhoN expression (Fig. S3) suggesting a lack of or inadequate Cascade induction. Therefore, all further experiments were done using pRAD1-based vector constructs with constitutive Cascade expression. *phoN* expression was assessed by spotting roughly equal number of cells on histochemical plates. Cells carrying pCRD2 or pRAD1 showed intense green coloration indicating the expression of *phoN,* while cells carrying pCRD2_PhoN_ showed no green coloration suggesting silencing of *phoN* in the latter (Fig. 2a). Zymogram for phosphatase expression showed presence of a 27 kDa band in protein extracts from cells carrying pCRD2_PhoN_ that was of lesser intensity than a similar sized band in cells carrying pCRD2 further confirming the silencing of the gene (Fig. 2b). Densitometry of the bands showed nine times lower phosphatase activity in protein extracted from pCRD2_PhoN_ compared to pCRD2. On the other hand, a native phosphatase that gave a band of approximately 130 kDa in zymogram showed equal intensity in protein extracts from both pCRD2_PhoN_ and pCRD2 carrying recombinants and served as a loading control (Fig. 2b; Fig. S4). The test and control strains were assessed for *phoN* expression in quantitative *p*NPP phosphatase assays and a 10-fold silencing efficiency was calculated (Fig. 2c).

### CRISPRi to downregulate *ssb*, a gene involved in radiation resistance

**Fig 3:**
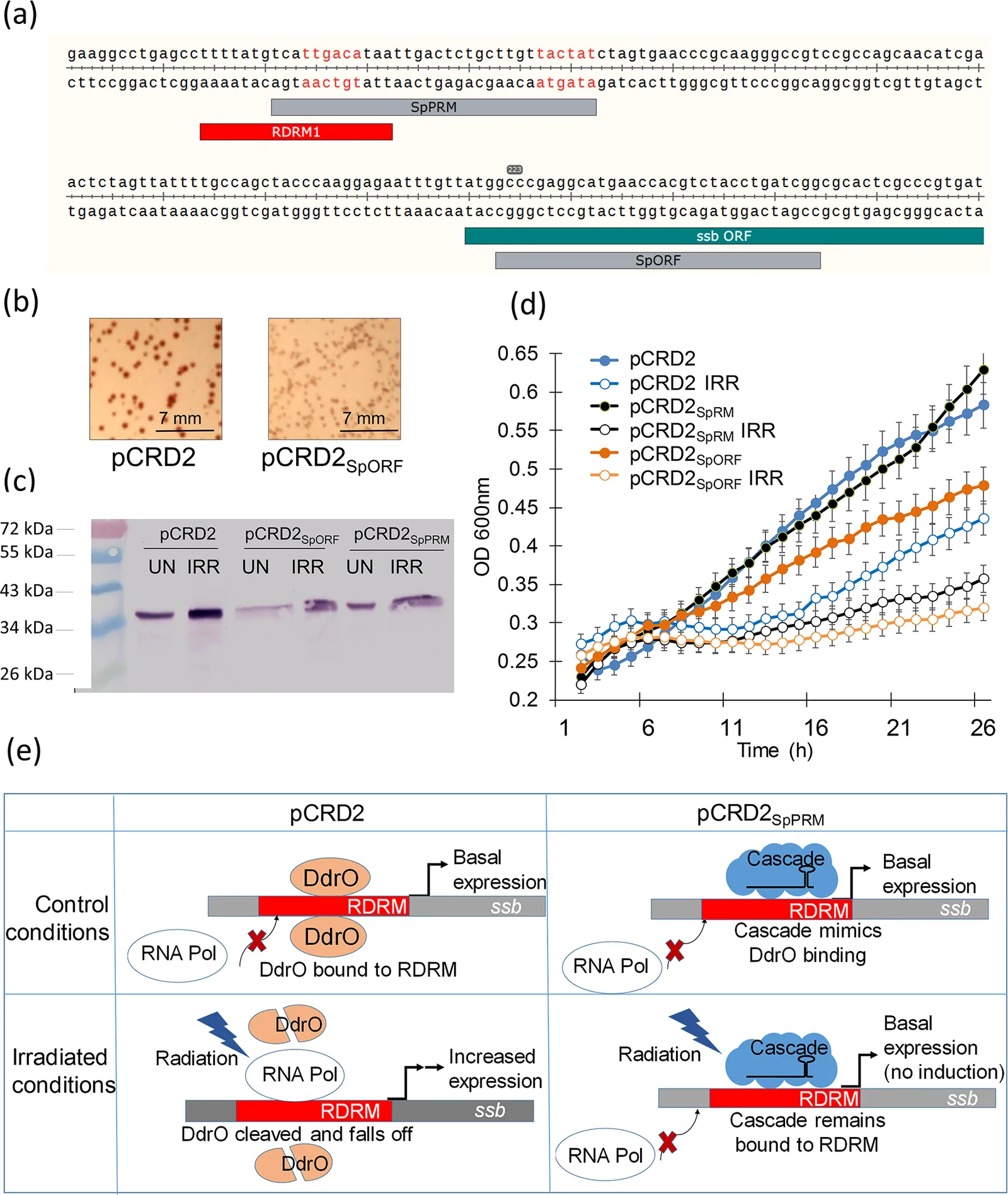
*ssb* Silencing at promoter and ORF using CRISPR-Cascade system. Two loci, one targeting the promoter (SpPRM) and the other targeting the ORF (SpORF) of the *ssb* using the CRISPR-Cascade system were identified. The −10 and −35 elements of the promoter are marked in red alphabets. The RDRM overlapping with the promoter is also marked. The illustration was made using Snapgene software (from Dotmatics, available at snapgene.com) (a). The plasmid, pCRD2 (expressing Cascade) or pCRD2_SpPRM_ (targeting *ssb* promoter) or pCRD2_SpORF_ (targeting *ssb* ORF) were transformed into *D. radiodurans*. Smaller sized colonies obtained on plating cells bearing pCRD2_SpORF_ compared to those bearing pCRD2 are shown (b). Western blot for detecting the Ssb protein in total protein extracted from irradiated (IRR) and unirradiated (UN) recombinant cells containing the indicated plasmids (c). Growth kinetics of recombinant cells during post-irradiation recovery (d). Mechanism of induction of *ssb* upon irradiation and its repression due to the binding of Cascade to RDRM (e).

The PhoN assay demonstrated that Cascade-based CRISPRi was functional in *D. radiodurans*. In order to establish it as a general gene silencing tool and to assess the physiological relevance of deinococcal gene silencing, we chose to knock down the native *ssb* gene. *ssb* is an essential gene in *D. radiodurans* that also plays an important role in radiation resistance (18). As in case of *phoN*, a spacer (SpORF) was targeted at the starting of the *ssb* ORF and cloned into pCRD2 (Fig. 3a) to generate pCRD2_SpORF_. *D. radiodurans* recombinants carrying pCRD2_SpORF_ gave rise to smaller colonies compared to those carrying pCRD2 (Fig. 3b). The expression of Ssb was analyzed on Western blot (Fig. 3c). Control cells carrying pCRD2 showed a basal level of expression. Upon irradiation, the Ssb levels showed a marked increase. Compared to the control cells, cells with pCRD2_SpORF_, showed much lower Ssb levels under unirradiated conditions confirming the knockdown of *ssb* expression. Upon irradiation, in these cells, Ssb levels did increase, but remained lower than irradiated control cells (Fig. 3c). Furthermore, under both control and irradiated conditions, cells containing pCRD2_SpORF_ showed poor growth compared to control cells (Fig. 3d), indicating effective knockdown of Ssb at physiologically relevant levels.

To investigate the effect of Cascade binding to a regulatory sequence element involved in radiation resistance, a spacer (SpPRM) was designed that targeted the promoter of the *ssb* while also overlapping with a Radiation and Desiccation Response Motif (RDRM) (Fig. 3a). In cells carrying pCRD2_SpPRM_, under unirradiated conditions, Ssb level was comparable to unirradiated control cells. However, in the same cells upon irradiation, the Ssb levels barely rose above the basal expression (Fig. 3c). Likewise, such cells showed normal growth under unirradiated conditions, but poor recovery from radiation (Fig. 3d). Normally, DdrO, a repressor of *ssb* expression remains bound to the RDRM sequences, keeping the Ssb expression to a basal level (41). Upon irradiation, the DdrO is cleaved, relieving repression of *ssb* and inducing its expression (42). Our results indicate that crRNA-guided binding of Cascade to RDRM releases *ssb* from DdrO-mediated regulation and prevents induction of *ssb* under irradiated conditions (Fig. 3e).

### Multiplexed gene silencing in *D. radiodurans*

**Fig 4:**
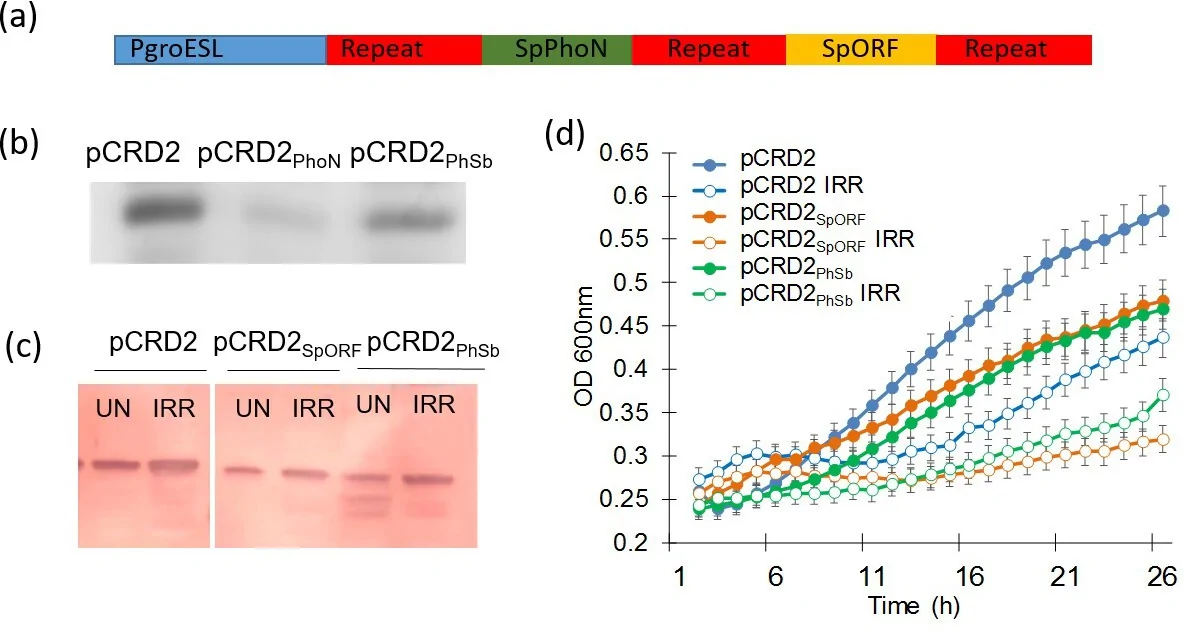
Silencing of *phoN* and *ssb* genes simultaneously in *D. radiodurans* using CRISPR-Cascade system. the cassette expressing the precrRNA in the pCRD2_PhSb_ plasmid, for targeting *phoN* and *ssb* using CRISPR-Cascade (a). Zymogram showing phosphatase silencing in recombinants, pCRD2_PhoN_ (targeting *phoN*) or pCRD2_PhSb_ (targeting *phoN* and *ssb*) or pCRD2 (control) (b). Western blot for detecting Ssb in total protein extracted from recombinant cells bearing pCRD2_SpORF_ (targeting *ssb* alone) or pCRD2_PhSb_ (targeting *phoN* and *ssb*) or pCRD2 (control) (c). Growth kinetics of recombinants under irradiated (IRR) and nonirradiated conditions.

Ability to target more than one gene simultaneously is one of the main advantages of the CRISPR system. To extend this feature of multiplexed silencing to *D. radiodurans*, a sequence coding for crRNAs targeting the *phoN* and *ssb* was synthesized (Fig. 4a) and cloned into the pCRD2 vector to generate pCRD2_PhSb_. Phosphatase silencing efficiency as observed from zymogram was lower in cells bearing pCRD2_PhSb_ than those bearing pCRD2_PhoN_ (Fig. 4b). Densitometric estimation showed 8.6-fold *phoN* downregulation in pCRD_PhoN_ cells compared to around 5.8-fold in pCRD_PhSb_ cells. pCRD2_PhSb_ carrying cells showed lower levels of Ssb (12-fold), which was similar to *ssb* downregulation in pCRD2_ORF_ cells (10.9-fold) (Fig. 4c). Growth defect and poor recovery from radiation in cells carrying pCRD2_PhSb_ were similar to that in pCRD2_ORF_ cells (Fig. 4d). Taken together, these results showed that two genes could be simultaneously knocked down in *D. radiodurans*.

## DISCUSSION

*D. radiodurans*, an organism of immense interest to researchers, did not have a system for carrying out targeted gene regulation. Further, multiple copies of the genome and a limited molecular toolkit made it difficult to study the organism. Recently, some systems for targeted gene regulation such as RNA interference and engineered transcription activator-like effector proteins and interference by CRISPR sequences have been exploited for regulation of expression in many organisms (25). While RNA interference is restricted to particular organisms, custom DNA-binding proteins are difficult to implement because of high cost associated with their designing and testing (21). Contrary to these, CRISPRi approach offers a simple and cost-effective tool principally applicable to all microorganisms for targeted gene regulation (20, 43).

The dearth of molecular tools in *D. radiodurans* has limited the progress in studying its radiation resistance mechanisms. For example, in well-worked out system of DdrO-based regulation of radiation response, it is still not known how PprI is activated (44). Not all the transcriptional regulators have been characterized in this microbe and the role of sRNAs in radiation response requires further investigation. Crosstalk among intricate regulatory networks that may play an important role in radiation resistance has not been well-studied owing to a lack of appropriate tools to regulate the expression of multiple genes in this microbe (45). The realization of full potential of this organism in industrial biotechnology has also been hampered by the absence of convenient genetic tools. In view of this, we report the addition of CRISPR-based gene silencing to the molecular toolkit available for this organism.

We used the type I-E system, as the type II-A dCas9 system was poorly tolerated in this organism, and problems with the use of the latter in microbes have earlier been reported due to dCas9 toxicity (37, 38, 46). This study demonstrates the application of Cascade-based system to knock down an assayable gene, *phoN*, as well as an essential gene, *ssb*. CRISPR-based gene silencing tools are ideally designed such that the Cas effector expression is under an inducible promoter. However, our attempt at placing the protein subunits of Cascade complex under the P_spac_ promoter of the vector pVHS559 for inducible gene expression in *D. radiodurans* did not yield effective silencing, perhaps due to poor expression, even under inducing conditions (30). The other inducible promoters characterized in *D. radiodurans* are induced by radiation which would confound results while studying radiation resistance in this organism. We therefore employed the strong constitutive promoter, P_groESL_, to drive Cascade expression.

As promoters in this organism are not very well characterized and are poorly predicted by bioinformatics tools (10), we targeted the start of the ORF regions to ascertain silencing efficiency, a feature that will have to be necessarily used for functional studies of hypothetical, novel, or poorly characterized genes. This is especially important in this organism considering the inability of *in silico* approaches to efficiently predict even strong deinococcal promoters (10). With the constitutive promoter P_groESL_ driving Cascade expression, the efficiency of knockdown of *phoN* obtained was around 90%, despite the multipartite genome in this organism.

Similarly, targeting the ORF of *ssb* resulted in low Ssb levels which in turn caused a growth defect under both irradiated and unirradiated conditions. Typically, modified CRISPR systems used for silencing of gene expression work best on targeting the promoter. Curiously, targeting the stretch of promoter which overlapped the RDRM sequence of *ssb* did not cause lower Ssb levels and likewise did not affect growth. The RDRM is normally bound by the DdrO, which regulates the expression of several genes upon irradiation. Binding to RDRM by DdrO keeps the *ssb* expression at a basal level by limiting access of RNA polymerase to the promoter (41). Upon irradiation, DdrO is cleaved by PprI, in turn causing de-repression of *ssb* expression (35, 42). Binding of Cascade to RDRM repressed *ssb*, mimicking DdrO and resulting in Ssb levels similar to control under unirradiated conditions (Fig. 3e). However, upon irradiation, unlike DdrO, Cascade remained bound to the RDRM, not permitting Ssb induction, resulting in severe growth defect. This showed the ability of the CRISPR-based gene regulation to characterize *cis*-elements involved in regulation of radiation response that could also be extended to promoter characterizations. An earlier study in *D. radiodurans* showed that *ssb* deletion from the chromosome and its expression from a plasmid at 42% of wild-type levels did not affect growth under normal conditions but resulted in radiation sensitivity. A further decrease in *ssb* expression to 28.5% of wild-type levels, also affected growth capabilities (18). However, unlike this study, the *ssb* expression from the plasmid was not from the native promoter and thus is not directly comparable with our system for irradiated conditions.

With Cascade-based CRISPRi, we demonstrate the ability to silence two genes simultaneously which could be expanded to target more genes by engineering the cassette expressing the crRNAs (47, 48). However, targeting two loci simultaneously resulted in lower knockdown efficiency for one of the two loci, compared to targeting of individual loci. This may indicate a titration effect of Cascade complexes getting distributed to different loci. Multiplexed gene regulation in *D. radiodurans* will enable the interrogation of several genes in a pathway or genes in different interacting pathways. This will aid the investigation of various multi-gene phenomena, such as radiation resistance apart, from finding use in manipulating substrate/product flux for metabolic engineering applications. In addition, CRISPRi screens will provide a high throughput method to probe gene networks. We anticipate that this easy-to-use gene silencing tool will facilitate the study of several interesting phenomena such as the role of sRNA and unique regulatory pathways in radiation resistance which have not been investigated in-depth in this organism.

## SUPPLEMENTAL MATERIAL

Supplemental information spectrum.05204-22-s0001.pdf Fig. S1 to S4 and Tables S1 to S4.

ASM does not own the copyrights to Supplemental Material that may be linked to, or accessed through, an article. The authors have granted ASM a non-exclusive, world-wide license to publish the Supplemental Material files. Please contact the corresponding author directly for reuse.
