# Supplementary material for: Effective gene silencing using type I–E CRISPR system in the multiploid, radiation-resistant bacterium Deinococcus radiodurans
Source: Microbiol Spectr. 2023 Sep 6;11(5):e05204-22. doi: 10.1128/spectrum.05204-22 (PMC10581213; doi:10.1128/spectrum.05204-22)
Supplement: Supplemental information — Fig. S1 to S4 and Tables S1 to S4. [file spectrum.05204-22-s0001.pdf]

Supplementary Information

Effective gene silencing using type I-E CRISPR system in the multiploid, radiation-resistant bacterium, *Deinococcus radiodurans*

Chitra S. Misra [a], Neha Pandey [a b], Deepti Appukuttan [c], Devashish Rath [a d #]

[a] Applied Genomics Section, Bio-Science Group, Bhabha Atomic Research Centre, Mumbai, India.

[b] Life Sciences, Mumbai University, Vidya Nagari, Kalina, Santacruz East, Mumbai, India.

[c] Chemical Engineering Department, IIT Bombay, Powai, Mumbai, India.

[d] Homi Bhabha National Institute, Anushaktinagar, Mumbai, India.

# Correspondence: Devashish Rath, Applied Genomics Section, Bio-Science Group, Bhabha Atomic Research Centre, Mumbai-400085. India. (devrath@barc.gov.in)

Running title: CRISPRi in *Deinococcus radiodurans*

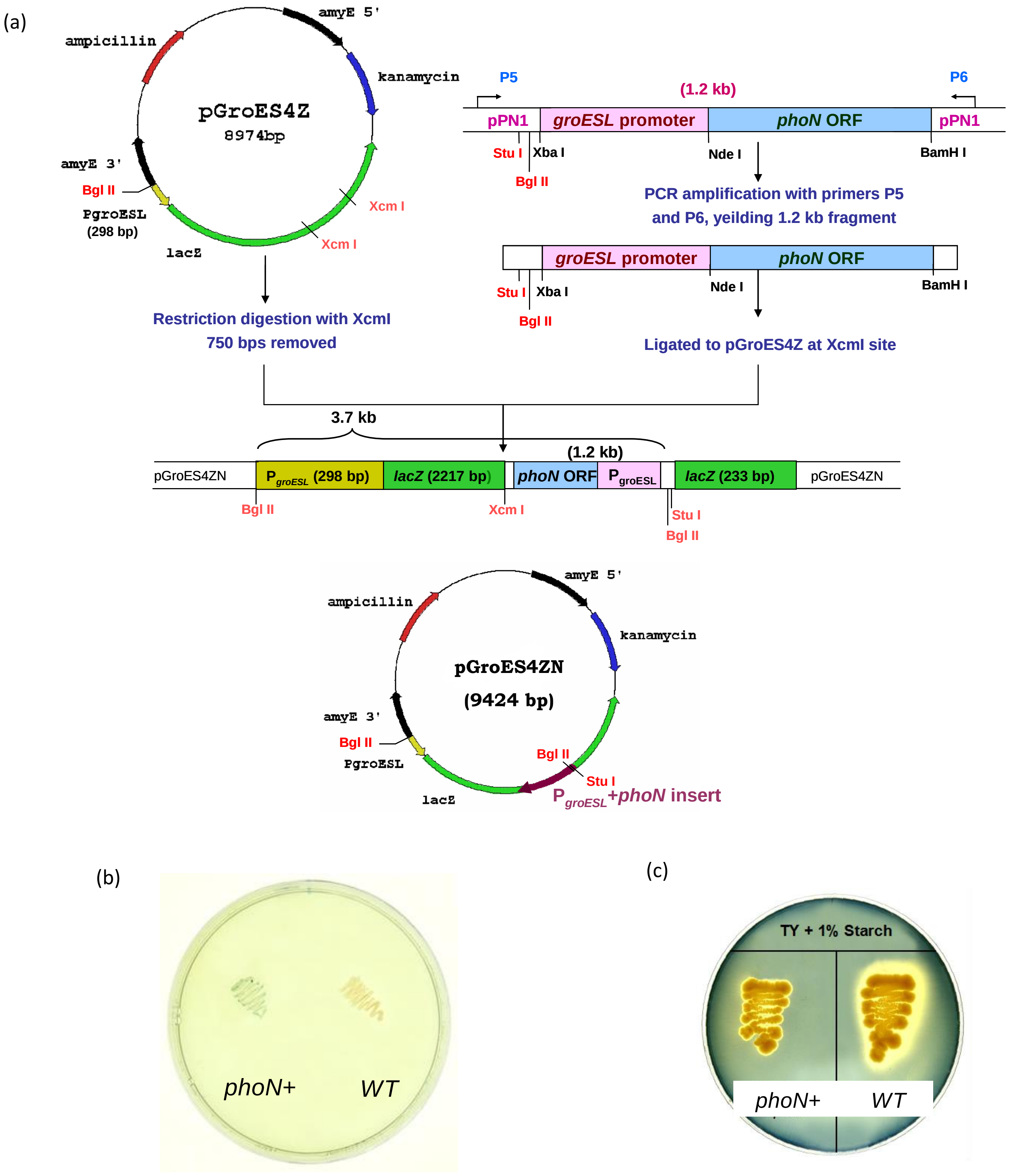

(a)
ampicillin
amyE 5'
kanamycin
pGroES4Z
8974bp
amyE 3'
Bgl II
PgroESL
(298 bp)
lacZ
Xcm I
Xcm I
Restriction digestion with XcmI
750 bps removed
P5
P6
(1.2 kb)
pPN1
groESL promoter
phoN ORF
pPN1
Stu I
Xba I
Bgl II
Nde I
BamH I
PCR amplification with primers P5
and P6, yeilding 1.2 kb fragment
groESL promoter
phoN ORF
Stu I
Xba I
Bgl II
Nde I
BamH I
Ligated to pGroES4Z at XcmI site
3.7 kb
(1.2 kb)
pGroES4ZN
P groESL (298 bp)
lacZ (2217 bp)
phoN ORF
P groESL
lacZ (233 bp)
pGroES4ZN
Bgl II
Xcm I
Stu I
Bgl II
ampicillin
amyE 5'
kanamycin
pGroES4ZN
(9424 bp)
amyE 3'
Bgl II
PgroESL
Bgl II
Stu I
lacZ
P groESL +phoN insert
(b)
phoN+
WT
(c)
TY + 1% Starch
phoN+
WT

Figure S1: Integration of *phoN* gene into deinococcal genome. (a) Cloning of *Salmonella typhi phoN* gene tagged to deinococcal *groESL* promoter into integration vector, pGroES4Z. The deinococcal *groESL* promoter along with the *phoN* ORF was PCR amplified using primers P5 and P6 from plasmid pPN1. The 1.2 kb PCR product was ligated to the XcmI digested pGroES4Z plasmid to yield pGroES4ZN. The relevant restriction enzyme sites are marked in red. Integration of the *phoN* gene into deinococcal chromosome was confirmed by (b) PhoN activity as observed on PDP-MG histochemical plate and (c) loss of starch hydrolysis observed on flooding a 1% starch agar plate with iodine.

graphical codon usage analyser 2.0

powered by www.mrgene.com $ 0.39 /bp 0.30 € /bp

www.gcua.de
created: 15.10.2019

Cas9
sequence derived from M. smegmatis

Codontable:
https://www.kazusa.or.jp/codon/cgi-bin/showcodon.cgi?species=1299&aa=1&style=N

Ordinate (y-axis): relative adaptiveness <20% <10%

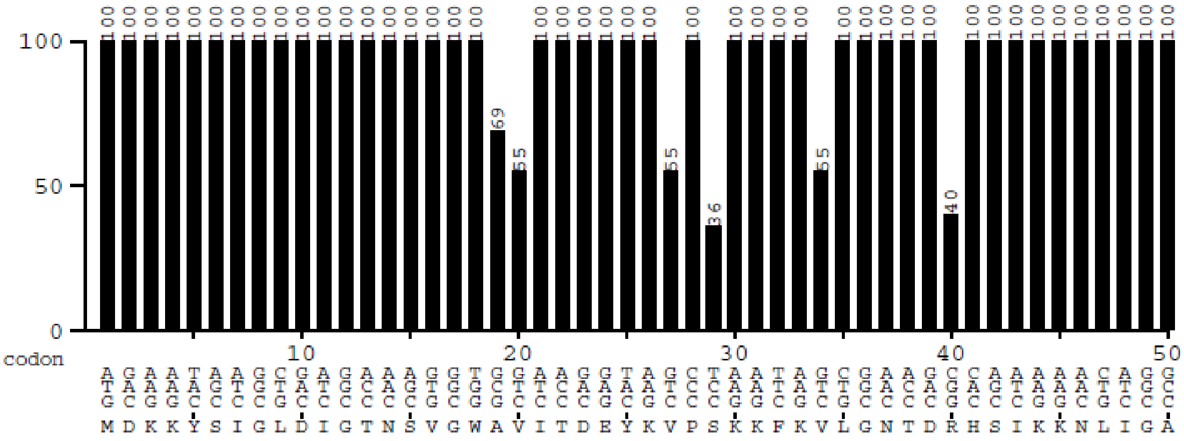

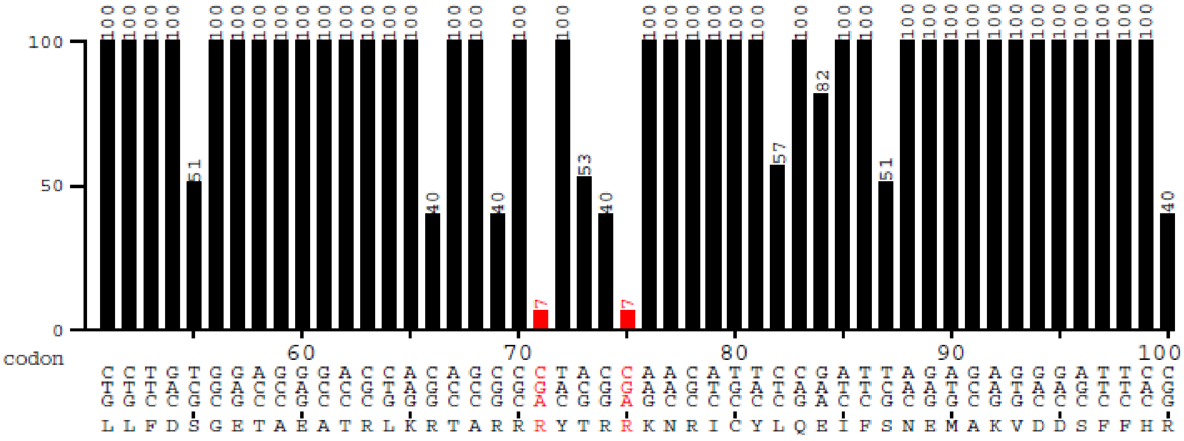

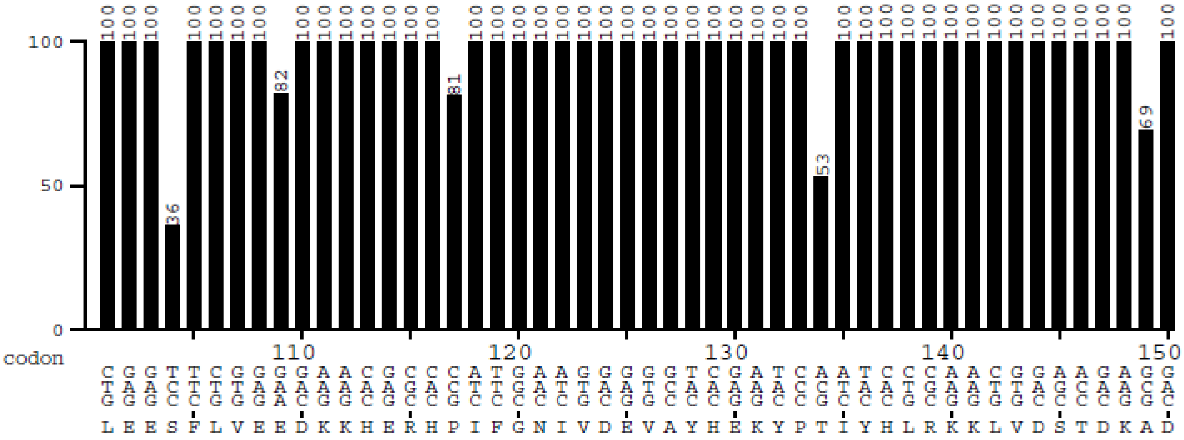

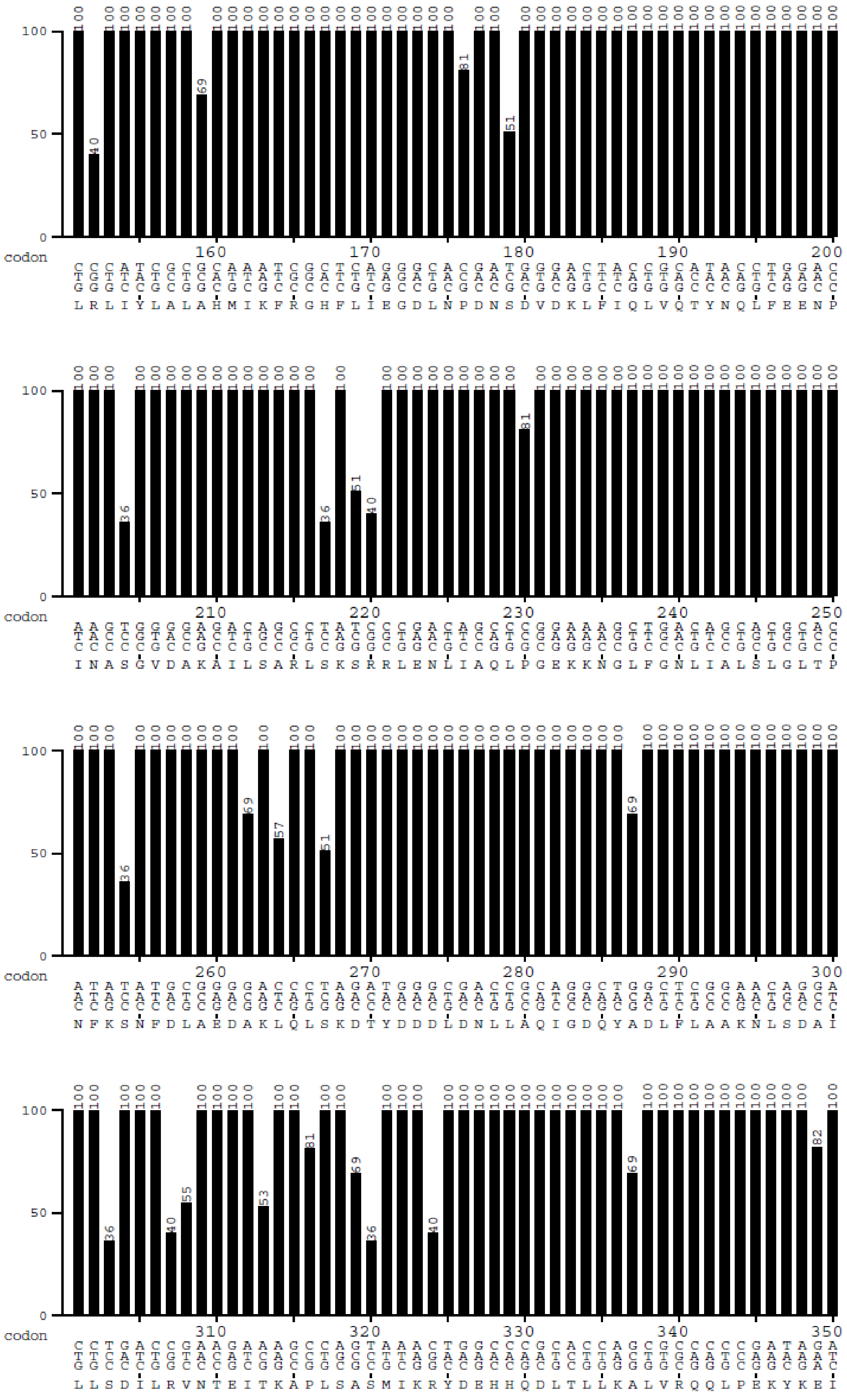

codon
160 170 180 190 200
codon
210 220 230 240 250
codon
260 270 280 290 300
codon
310 320 330 340 350

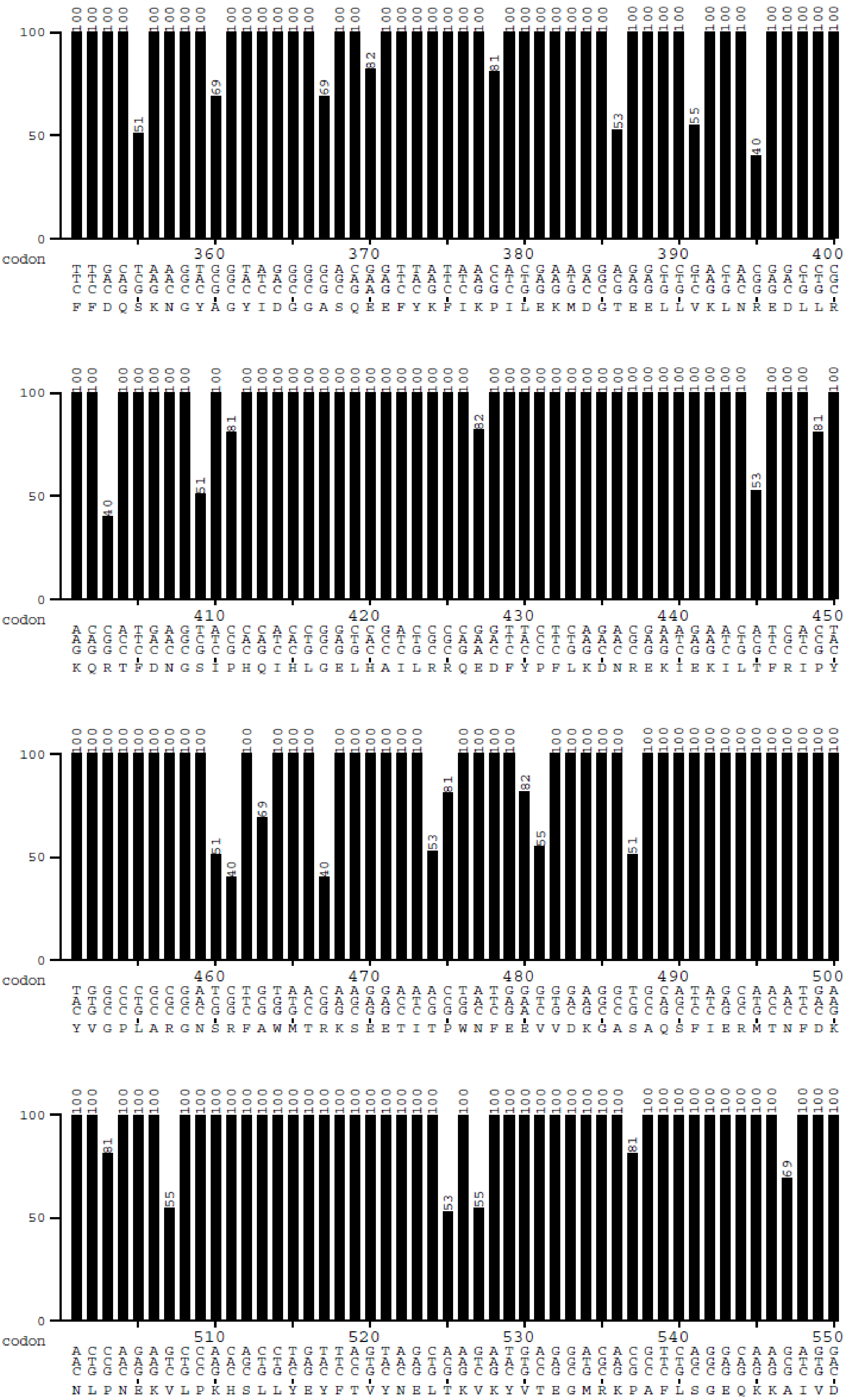
codon
360 370 380 390 400
codon
410 420 430 440 450
codon
460 470 480 490 500
codon
510 520 530 540 550

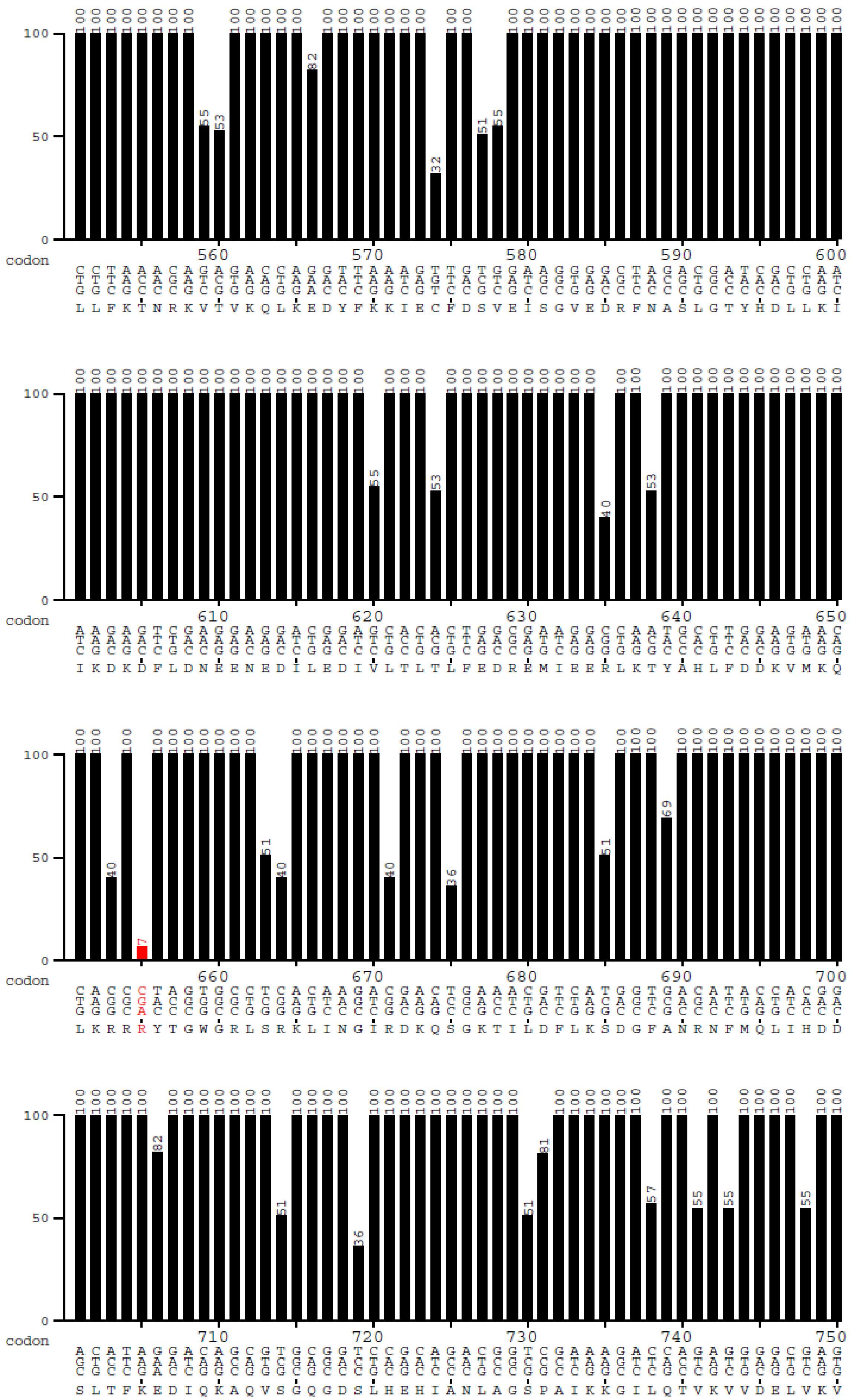
100
50
0
codon
560 570 580 590 600
L L F K T N R K V T V K Q L K E D Y F K K I E C F D S V E I S G V E D R F N A S L G T Y H D L L K I
610 620 630 640 650
I K D K D F L D N E E N E D I L E D I V L T L T L F E D R E M I E E R L K T Y A H L F D D K V M K Q
660 670 680 690 700
L K R R R Y T G W G R L S R K L I N G I R D K Q S G K T I L D F L K S D G F A N R N F M Q L I H D D
710 720 730 740 750
S L T F K E D I Q K A Q V S G Q G D S L H E H I A N L A G S P A I K K G I L Q T V K V V D E L V K V

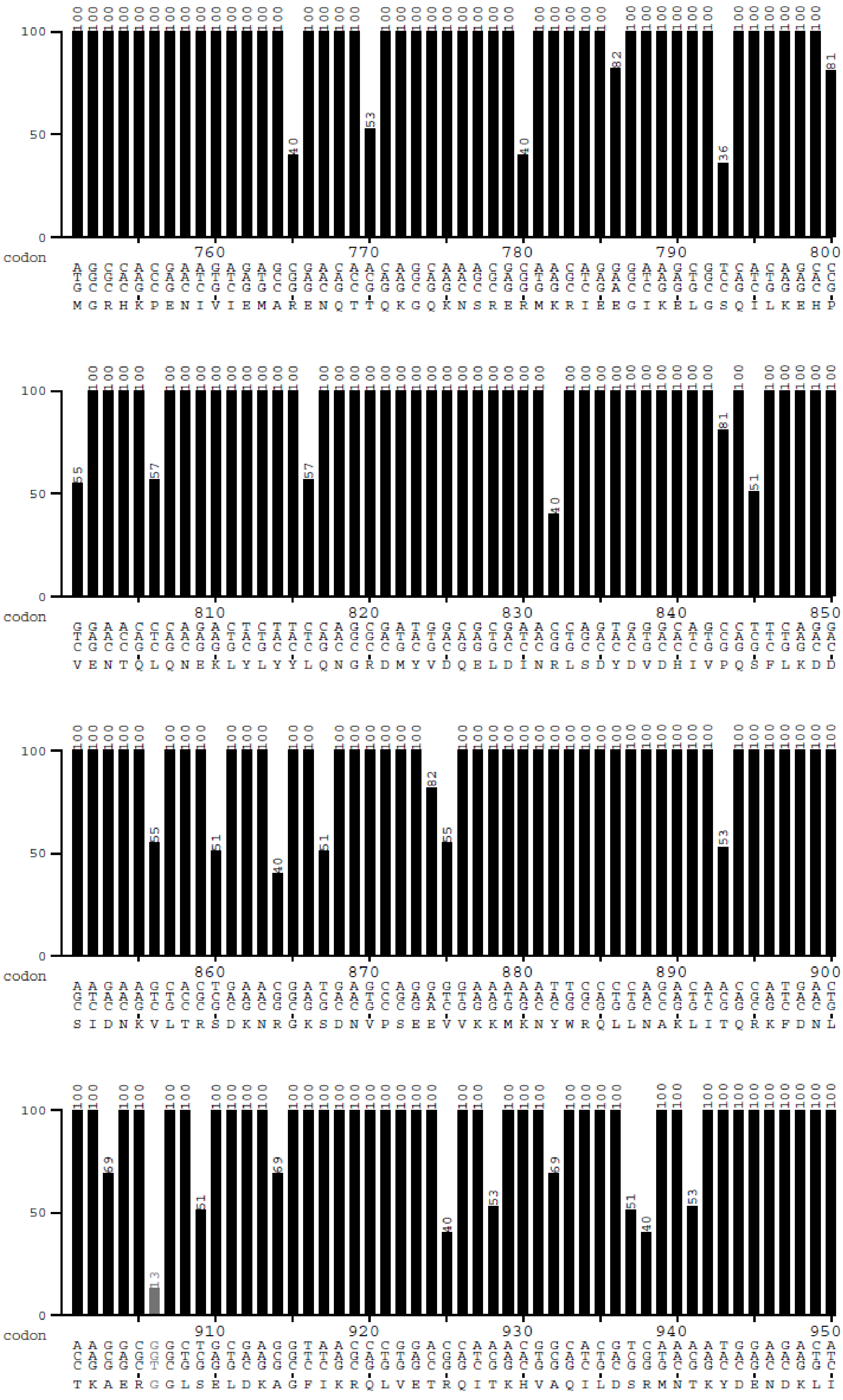

100
50
0
codon
760 770 780 790 800
M G R H K P E N I V I E M A R E N Q T T Q K G Q K N S R E R M K R I E E G I K E L G S Q I L K E H P
810 820 830 840 850
V E N T Q L Q N E K L Y L Y Y L Q N G R D M Y V D Q E L D I N R L S D Y D V D H I V P Q S F L K D D
860 870 880 890 900
S I D N K V L T R S D K N R G K S D N V P S E E V V K K M K N Y W R Q L L N A K L I T Q R K F D N L
910 920 930 940 950
T K A E R G G L S E L D K A G F I K R Q L V E T R Q I T K H V A Q I L D S R M N T K Y D E N D K L I

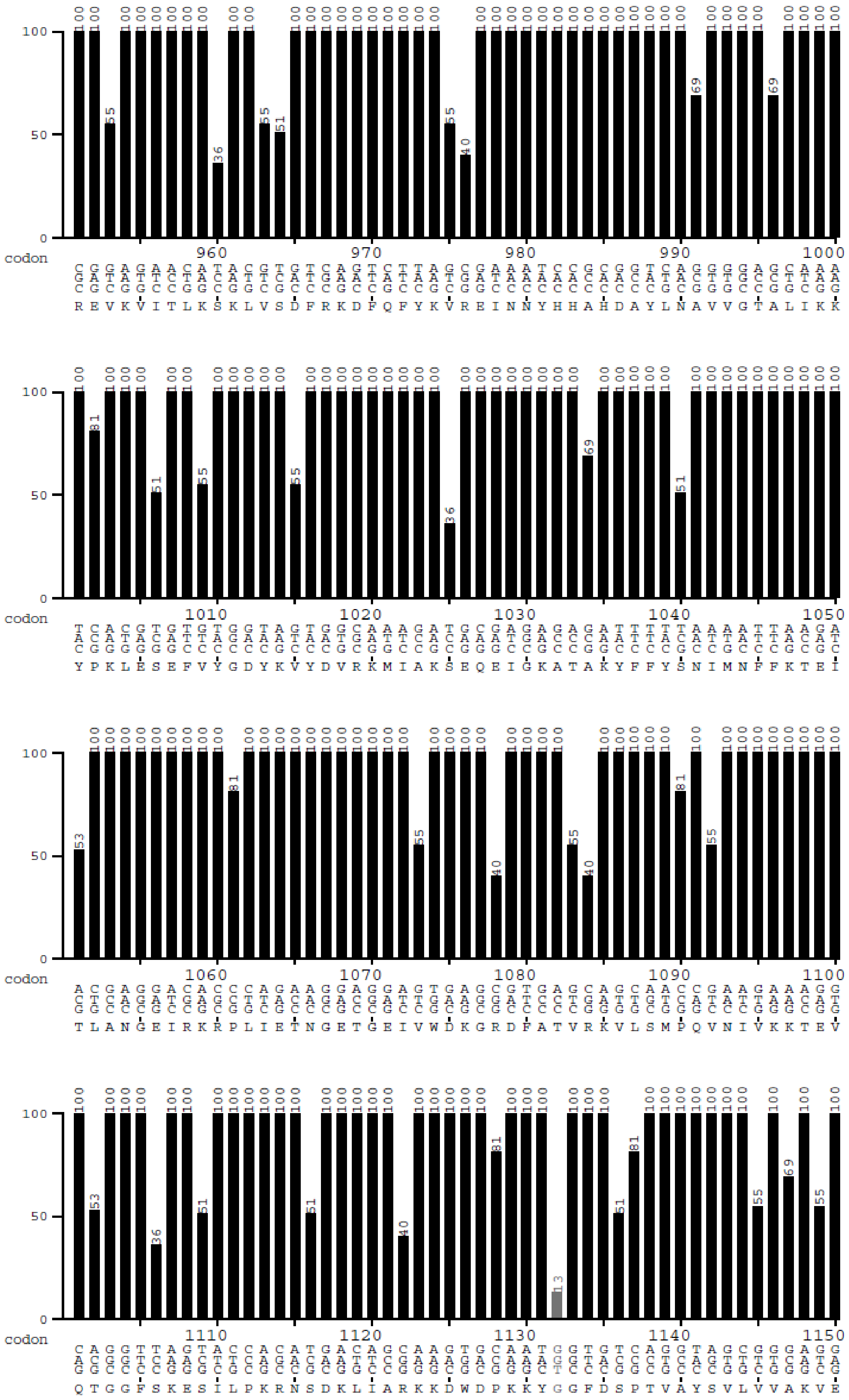
codon
R E V K V I T L K S K L V S D F R K D F Q F Y K V R E I N N Y H H A H D A Y L N A V V G T A L I K K
Y P K L E S E F V Y G D Y K V Y D V R K M I A K S E Q E I G K A T A K Y F F Y S N I M N F F K T E I
T L A N G E I R K R P L I E T N G E T G E I V W D K G R D F A T V R K V L S M P Q V N I V K K T E V
Q T G G F S K E S I L P K R N S D K L I A R K K D W D P K K Y G G F D S P T V A Y S V L V V A K V E

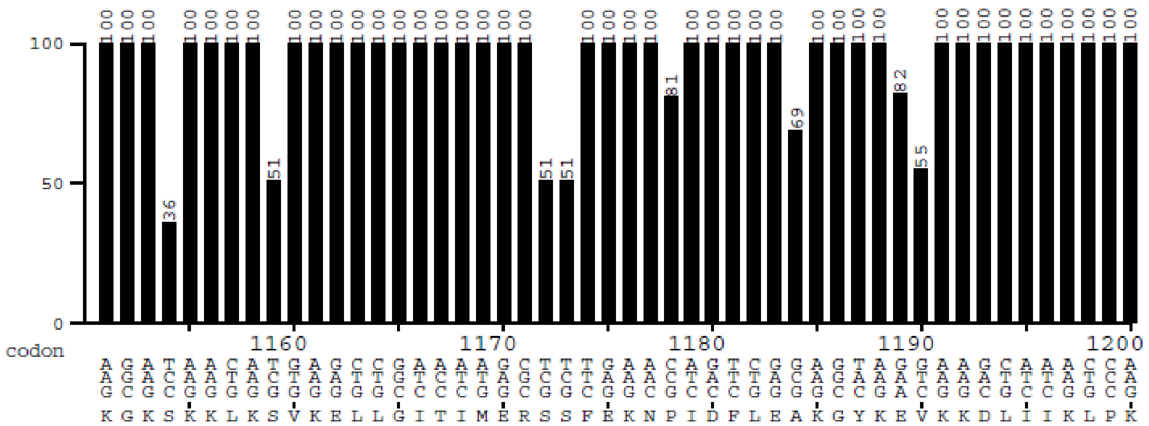
100
50
0
codon
1160 1170 1180 1190 1200
K G K S K K L K S V K E L L G I T I M E R S S F E K N P I D F L E A K G Y K E V K K D L I I K L P K

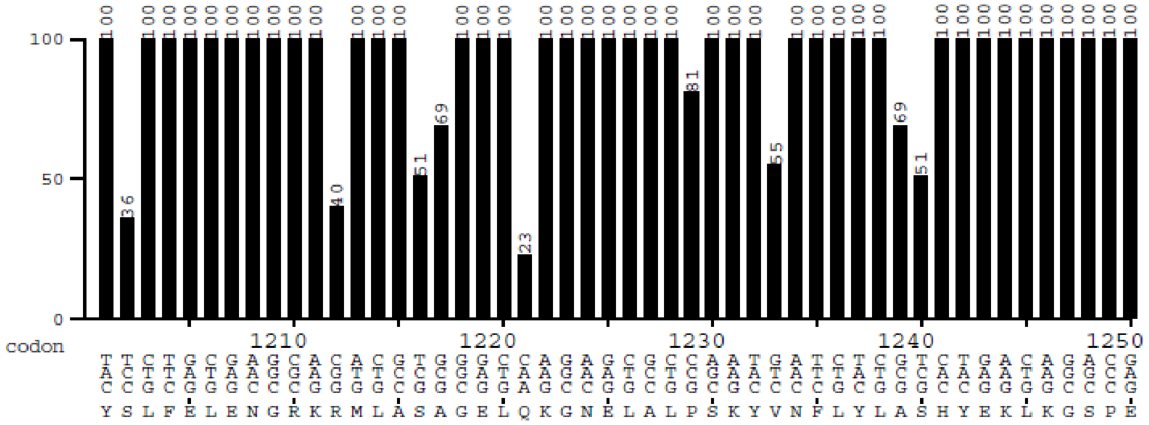
100
50
0
codon
1210 1220 1230 1240 1250
Y S L F E L E N G R K R M L A S A G E L Q K G N E L A L P S K Y V N F L Y L A S H Y E K L K G S P E

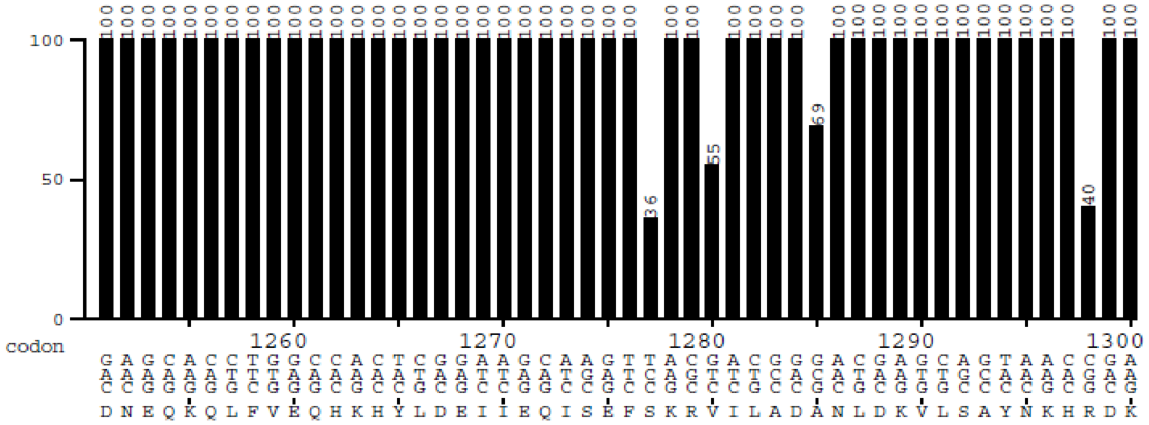
100
50
0
codon
1260 1270 1280 1290 1300
D N E Q K Q L F V E Q H K H Y L D E I I E Q I S E F S K R V I L A D A N L D K V L S A Y N K H R D K

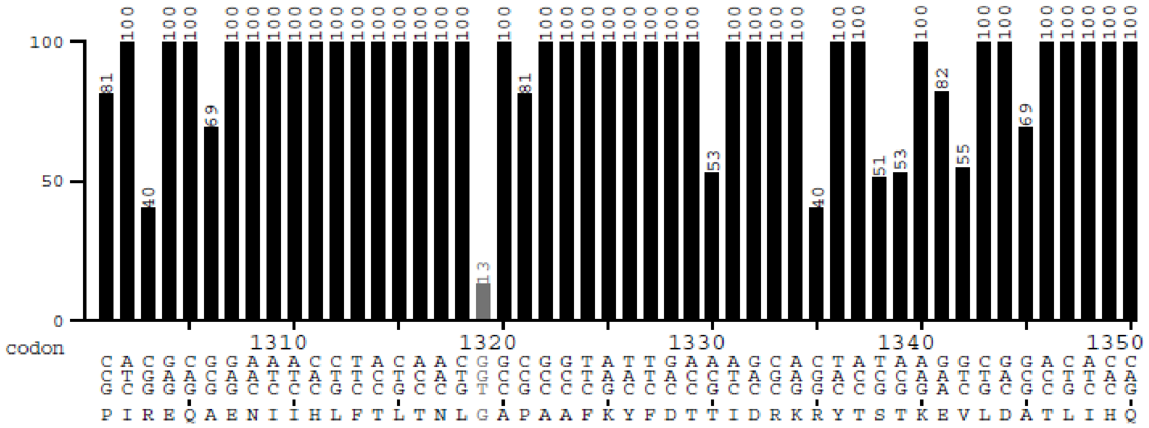
100
50
0
codon
1310 1320 1330 1340 1350
P I R E Q A E N I I H L F T L T N L G A P A A F K Y F D T T I D R K R Y T S T K E V L D A T L I H Q

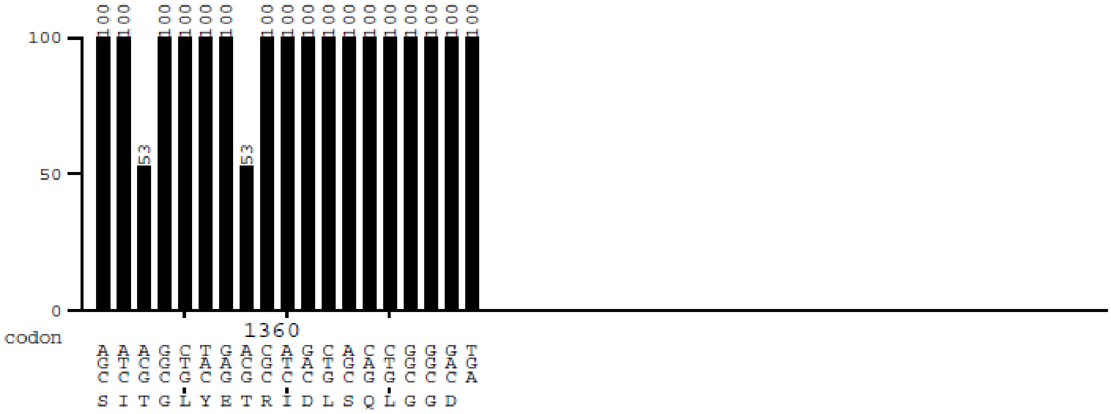

Figure S2. Analysis of expression of *cas9*, optimized for *M. smegmatis* in *D. radiodurans* with Graphical Codon Analyzer, based on codon usage in the latter. The y axis indicates relative adaptiveness value, described as the ratio of the frequency of given codon to that of the most abundant codon for same amino acid multiplied by 100. Bars with value <20 are marked in grey, while bars with value <10 are marked in red. Black bars have a value >20, and codons corresponding to them are expected to be present at high frequency in the organism. The abundance of black bars in this analysis indicates a likelihood of good expression of the given gene. D10A and H840A mutations were introduced into Cas9 to convert it to dCas9. The introduced alanine codon, GCC shows a high frequency of usage in *D. radiodurans*.

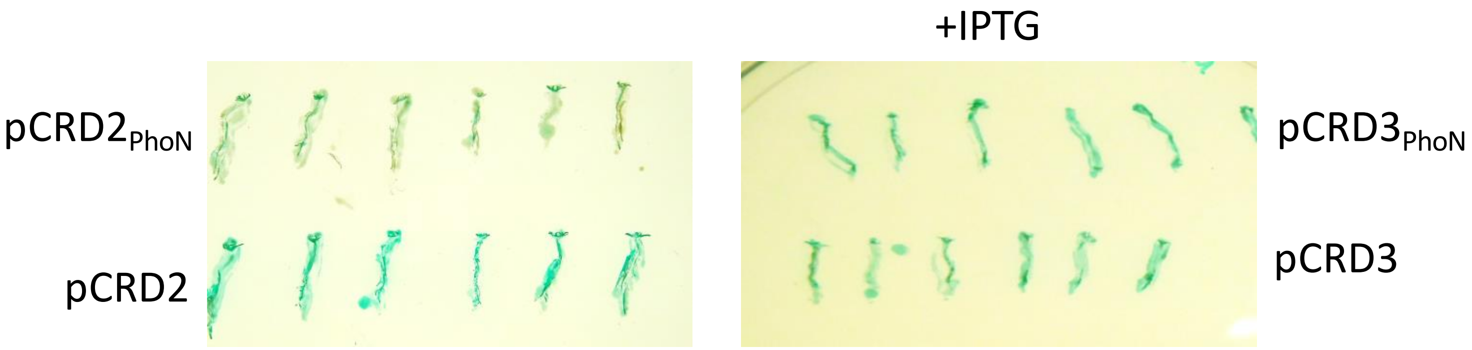

Figure S3. *phoN* silencing by constitutive or inducible expression of Cascade. Recombinants carrying pCRD2/pCRD2$_{PhoN}$ (constitutive expression of Cascade) or pCRD3/pCRD3$_{PhoN}$ (inducible expression of Cascade) were streaked on media containing PDP-MG. IPTG was added in case of pCRD3 for induction of Cascade.

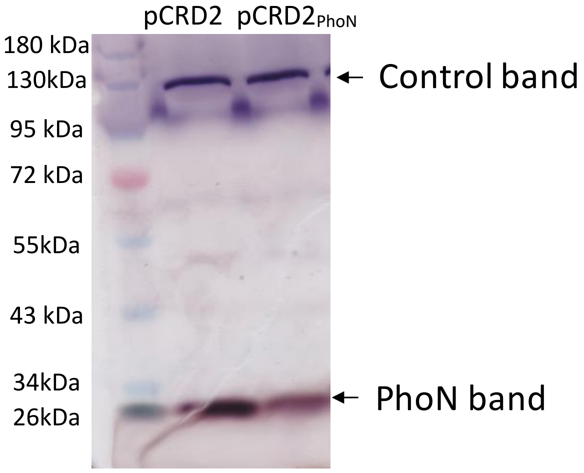

Figure S4. Zymogram to show silencing of PhoN, with the band for a native phosphatase serving as protein normalization control.

Supplementary Tables

Table S1: Plasmids and strains used in study

**Strains**	Description	Source/Reference
Escherichia coli	JM109 strain	Lab strain
Escherichia coli	DH5α strain	Lab strain
Deinococcus radiodurans	*R1* strain	Lab strain
Deinococcus radiodurans R1 phoN+	*D. radiodurans* with the P_{groESL}+*phoN* gene integrated into its genome	This study
**Plasmids**		
pRAD1	*E. coli-D. radiodurans* shuttle vector; Ap^r Cm^r; 6.28 kb	Meima et al., 2001
pPN1	pRAD1 containing *S. enterica* serovar Typhi *phoN* gene with deinococcal *groESL* promoter	Appukuttan et al., 2006
pCRD1	pRAD1 containing Cas9 under P_{groESL} promoter	This study
pCRD2	pRAD1 containing both Cascade genes and crRNA under P_{groESL} promoter	This study
pVHS559	*E. coli-D. radiodurans* shuttle vector; $Spec^r$ Cm^r; 9.9 kb	Charaka et al., 2012
pCRD3	pVHS559 containing Cas9 under P_{spac} promoter	This study
$pCRD2_{PhoN}$	pCRD2 with crRNA targeting PhoN	This study
$pCRD3_{PhoN}$	pCRD3 with crRNA targeting PhoN	This study
$pCRD2_{SpORF}$	pCRD2 with crRNA targeting *ssb* ORF	This study
$pCRD2_{SpPRM}$	pCRD2 with crRNA targeting *ssb* promoter	This study
$pCRD2_{PhSb}$	pCRD2 with crRNA targeting the ORF of both, *phoN* and *ssb*.	This study
pSTKT-dCas9	*E. coli-Mycobacterium* shuttle vector with Cas9 codon optimized for *Mycobacterium* cloned in it	Sodani et al., 2023
pGroES4Z	Replicative plasmid in *E. coli*, for integrating genes into the *amyE* locus of the chromosome in *D. radodurans*; Ap^r, Kan^r	Meima et al., 2006
pGroES4ZN	pGroES4ZN carrying P_{groESL} and *phoN* cloned between the *amyE* flanks for integration into the deinococcal genome.	This study

Table S2:

Sequence of Cascade codon optimised for deinococcal expression:

CAT**atg**
AACCTGCTGATCGACAACTGGATTCCCGTGCGCCCGCGCAACGGTGGCAAGGTGCAGATCATCAAC
CTGCAAAGCCTGTACTGCTCGCGCGACCAGTGGCGCCTGAGCCTGCCCCGCGACGACATGGAGCTG
GCCGCGCTGGCCCTGCTGGTGTGCATCGGCCAGATCATCGCCCCCGCCAAGGACGACGTGGAGTTC
CGCCACCGCATCATGAACCCGCTGACCGAGGACGAGTTCCAGCAGCTGATCGCGCCGTGGATCGAC
ATGTTCTACCTGAACCACGCCGAGCACCCCTTCATGCAGACCAAGGGCGTGAAGGCGAACGACGTG
ACCCCGATGGAAAAGCTGCTGGCCGGCGTGAGCGGCGCCACCAACTGCGCCTTCGTGAACCAGCCT
GGCCAGGGCGAGGCCCTGTGCGGCGGCTGCACCGCCATCGCGCTGTTCAACCAGGCCAACCAGGCC
CCTGGCTTCGGCGGCGGCTTCAAGAGCGGCCTGCGCGGCGGCACCCCGGTGACCACCTTCGTGCGC
GGCATCGACCTGCGCAGCACCGTGCTGCTGAACGTGCTGACCCTGCCCCGCCTCCAGAAGCAGTTCC
CGAACGAGAGCCACACCGAAAACCAGCCCACCTGGATCAAGCCGATCAAGAGCAACGAGAGCATCC
CCGCGAGCAGCATCGGCTTCGTGCGCGGCCTGTTCTGGCAGCCGGCCCACATCGAACTGTGCGACC
CGATCGGCATCGGCAAGTGCAGCTGCTGCGGCCAGGAGAGCAACCTGCGCTACACCGGCTTCCTGA
AGGAAAAGTTCACCTTCACCGTGAACGGCCTGTGGCCCCACCCGCACAGCCCCTGCCTGGTGACCGT
GAAGAAGGGCGAGGTGGAGGAAAAGTTCCTGGCCTTCACCACCAGCGCCCCCAGCTGGACCCAGA
TCAGCCGCGTGGTGGTGGACAAGATCATCCAGAACGAAAACGGCAACCGCGTGGCCGCGGTGGTG
AACCAGTTCCGCAACATCGCGCCCCAGAGCCCGCTGGAGCTGATCATGGGCGGCTACCGCAACAAC
CAGGCCAGCATCCTGGAACGCCGCCACGACGTGCTGATGTTCAACCAGGGCTGGCAGCAGTACGGC
AACGTGATCAACGAGATCGTGACCGTGGGCCTGGGCTACAAGACCGCCCTGCGCAAGGCCCTGTAC
ACCTTCGCGGAAGGCTTCAAGAACAAGGACTTCAAGGGCGCGGGCGTGAGCGTGCACGAAACCGC
CGAGCGCCACTTCTACCGCCAGAGCGAGCTGCTGATCCCCGACGTGCTGGCCAACGTGAACTTCAGC
CAGGCCGACGAGGTGATCGCCGACCTGCGCGACAAGCTGCACCAGCTGTGCGAAATGCTGTTCAAC
CAGAGCGTGGCCCCCTACGCCCACCACCCGAAGCTGATCAGCACCCTGGCCCTGGCGCGCGCCACC
CTGTACAAGCACCTGCGCGAGCTGAAGCCG ca**aggagg** gccCAGCa**at g**gc

tgaC
GAGATCGACGCGATGGCCCTGTACCGCGCCTGGCAGCAGCTGGACAACGGCAGCTGCGCCCAGATC
CGCCGCGTGAGCGAGCCGGACGAACTGCGCGACATCCCCGCCTTCTACCGCCTGGTGCAGCCCTTC
GGCTGGGAAAACCCGCGCCACCAGCAGGCCCTGCTGCGCATGGTGTTCTGCCTGAGCGCGGGCAA
GAACGTGATCCGCCACCAGGACAAGAAGAGCGAGCAGACCACCGGCATCAGCCTGGGCCGCGCCC
TGGCCAACAGCGGCCGGATCAACGAACGTCGCATCTTCCAGCTGATCCGCGCGGACCGCACCGCGG
ACATGGTGCAGCTGCGCCGCCTGCTGACCCACGCGGAGCCGGTGCTGGACTGGCCCCTGATGGCCC
GCATGCTGACCTGGTGGGGCAAGCGCGAGCGCCAGCAGCTGCTGGAGGACTTCGTGCTGACCACC
AACAAGAACGCG

taaggaGGcctt tct

atg
AGCAACTTCATCAACATCCACGTGCTGATCAGCCACAGCCCCAGCTGCCTGAACCGCGACGACATGA
ACATGCAGAAGGACGCCATCTTCGGTGGCAAGCGTCGCGTGCGCATCAGCAGCCAGAGCCTGAAGC
GCGCCATGCGCAAGAGCGGCTACTACGCGCAGAACATCGGCGAAAGCAGCCTGCGCACCATCCACC
TGGCCCAGCTGCGCGACGTGCTGCGCCAGAAGCTGGGCGAGCGCTTCGACCAGAAGATCATCGACA

AGACCCTGGCCCTGCTGAGCGGCAAGAGCGTGGACGAGGCGGAAAAGATCAGCGCGGACGCGGT
GACCCCGTGGGTGGTGGGCGAGATCGCCTGGTTCTGCGAACAGGTGGCGAAGGCCGAGGCGGACA
ACCTGGACGACAAGAAGCTGCTGAAGGTGCTGAAAGAGGACATCGCCGCGATCCGCGTGAACCTG
CAACAGGGCGTGGACATCGCCCTGAGCGGCCGGATGGCCACCAGCGGCATGATGACCGAGCTGGG
CAAGGTGGACGGCGCCATGAGCATCGCCCACGCGATCACCACCCACCAGGTGGACAGCGACATCGA
CTGGTTCACCGCCGTGGACGACCTGCAAGAACAGGGCAGCGCCCACCTGGGCACCCAGGAGTTCAG
CAGCGGCGTGTTCTACCGCTACGCCAACATCAACCTGGCCCAGCTCCAGGAAAACCTGGGCGGCGC
CAGCCGCGAGCAGGCCCTGGAAATCGCCACCCACGTGGTGCACATGCTGGCCACCGAGGTGCCCGG
CGCCAAGCAGCGCACCTACGCCGCCTTCAACCCGGCGGACATGGTGATGGTGAACTTCAGCGACAT
GCCCCTGAGCATGGCCAACGCGTTCGAGAAGGCCGTGAAGGCGAAGGACGGCTTCCTCCAGCCGA
GCATCCAGGCCTTCAACCAGTACTGGGACCGCGTGGCCAACGGCTACGGCCTGAACGGCGCTGCCG
CCCAGTTCAGCCTGAGCGACGTGGACCCCATCACCGCCCAGGTGAAGCAGATGCCGACCCTGGAAC
AGCTGAAGAGCTGGGTGCGCAACAACGGCGAGGCG

TgaGGAGGCCTT TCT

atg
CGCAGCTACCTGATCCTGCGCCTGGCCGGCCCCATGCAGGCGTGGGGCCAGCCGACCTTCGAGGGC
ACCCGCCCCACCGGCCGCTTCCCGACCCGCAGCGGCCTGCTGGGCCTGCTGGGCGCCTGCCTGGGC
ATCCAGCGCGACGACACCAGCAGCCTCCAGGCCCTGAGCGAGAGCGTGCAGTTCGCCGTGCGCTGC
GACGAACTGATCCTGGACGACCGCCGCGTGAGCGTGACCGGCCTGCGCGACTACCACACCGTGCTG
GGCGCCCGCGAGGACTACCGCGGCCTGAAGAGCCACGAAACCATCCAGACCTGGCGCGAATACCTG
TGCGACGCCAGCTTCACCGTGGCCCTGTGGCTGACCCCGCACGCCACGATGGTGATCAGCGAGCTG
GAAAAGGCGGTGCTGAAGCCCCGCTACACCCCGTACCTGGGCCGCCGCAGCTGCCCCCTGACCCAC
CCGCTGTTCCTGGGCACCTGCCAGGCCAGCGACCCGCAGAAGGCGCTGCTGAACTACGAGCCCGTG
GGCGGCGACATCTACAGCGAGGAAAGCGTGACCGGCCACCACCTGAAGTTCACCGCCCGCGACGA
GCCCATGATCACCCTGCCCCGCCAGTTCGCGAGCCGCGAATGGTACGTGATC aaagg aggCatgg **at**
gtatctcag

t aaa
GTGATCATCGCCCGTGCCTGGAGCCGCGACCTGTACCAGCTGCACCAGGGCCTGTGGCACCTGTTCC
CCAACCGCCCCGACGCGGCGCGCGACTTCCTGTTCCACGTGGAGAAGCGCAACACCCCGGAAGGCT
GCCACGTGCTGCTCCAGAGCGCCCAGATGCCGGTGAGCACCGCCGTGGCGACCGTGATCAAGACCA
AGCAGGTGGAGTTCCAGCTCCAGGTGGGCGTGCCCCTGTACTTCCGCCTGCGCGCCAACCCGATCA
AGACCATCCTGGACAACCAGAAGCGCCTGGACAGCAAGGGCAACATCAAGCGCTGCCGCGTGCCCC
TGATCAAGGAAGCCGAACAGATCGCGTGGCTCCAGCGCAAGCTGGGCAACGCGGCGCGCGTGGAG
GACGTGCACCCCATCAGCGAACGCCCGCAGTACTTCAGCGGCGACGGCAAGAGCGGCAAGATCCA
GACCGTGTGCTTCGAAGGCGTGCTGACCATCAACGACGCCCCGGCCCTGATCGACCTGGTGCAGCA
GGGCATCGGCCCTGCCAAGAGCATGGGCTGCGGCCTGCTGAGCCTGGCGCCGCTG **tga**

Red alphabets – Start /stop codons, Highlighted sequence – Shine Dalgarno sequence

Table S3: Oligos used in study

oligo Name	5'-3' Sequence	Description
Pgrocas-f	CGTCATATGGGGTCCTCCTGTGAGTGAG	For cloning PgroESL into pRAD1
Pgrocas-r	AATCGGATCCCATGTTCAGGGATGGAAGCAC	For cloning PgroESL into pRAD1
CorrPhoNSpa-f	TCGAGAGTTCCCCGCGCCAGCGGGGATAAACCGAAA AGTCGTTATTTACTATTTTT TCTACCACTGAG TTCCCCGCGCCAGCGGGGATAAACCGAGCT	Spacer for targeting PhoN
CorrPhoNSpa-r	CGGTTTATCCCCGCTGGCGCGGGGAACTCAGTGGTA GAAAAAAATAGTAAATAACGACTTTTCGGTT TATCCCCGCTGGCGCGGGGAACTC	Spacer for targeting PhoN
SpaSsb1f	TCGAGAGTTCCCCGCGCCAGCGGGGATAAACCGTCA TTGACATAATTGACTCTGCTTGTTACTAT GAGTTCCCCGCGCCAGCGGGGATAAACCGAGCT	Spacer for targeting promoter of Ssb
SpaSsb1r	CGGTTTATCCCCGCTGGCGCGGGGAACTCATAGTAAC AAGCAGAGTCAATTATGTCAATGA CGGTTTATCCCCGCTGGCGCGGGGAACTC	Spacer for targeting promoter of Ssb
SpaSsb3f	TCGAGAGTTCCCCGCGCCAGCGGGGATAAACCGGCC CGAGGCATGAACCACGTCTACCTGATCGG GAGTTCCCCGCGCCAGCGGGGATAAACCGAGCT	Spacer for targeting ORF of Ssb
SpaSsb3r	CGGTTTATCCCCGCTGGCGCGGGGAACTCCCGATCAG GTAGACGTGGTTCATGCCTCGGGC CGGTTTATCCCCGCTGGCGCGGGGAACTC	Spacer for targeting ORF of Ssb
P5	GGAGCGGATAACAATTTCACACA	For integration of P_{groESL}+*phoN* construct into *Deinococcus* genome
P6	AACGCGGCTGCAAGAATGGTA	For integration of P_{groESL}+*phoN* construct into *Deinococcus* genome
Amy-1	CGTATGCCTCACCCTGACATC	Diagnostic primers to confirm *phoN* integration in *D. radiodurans*
Amy-2	AGATTTTGAGACACAACGTG	Diagnostic primers to confirm *phoN* integration in *D. radiodurans*
Amy-3	CTCCTGAGATCTTCCCTGCAG	Diagnostic primers to confirm *phoN* integration in *D. radiodurans*
Amy-4	GATGAGGGAGCAAGTCAGAC	Diagnostic primers to confirm *phoN* integration in *D. radiodurans*

Table S4: Phosphatase activity in different strains

Strain	Plasmid	Phosphatase activity (nmol *p*-NP released/mg protein/min)
D. radiodurans	pRAD1	30 ± 5
D. radiodurans	pPN1	190 ±10
D. radiodurans	-	32 ± 3
D. radiodurans phoN+	-	65 ± 5
